# An Ensemble-of-Classifiers Based Approach for Early Diagnosis of Alzheimer's Disease: Classification Using Structural Features of Brain Images

**DOI:** 10.1155/2014/862307

**Published:** 2014-09-09

**Authors:** Saima Farhan, Muhammad Abuzar Fahiem, Huma Tauseef

**Affiliations:** Department of Computer Science, Lahore College for Women University, Jail Road, Lahore 54000, Pakistan

## Abstract

Structural brain imaging is playing a vital role in identification of changes that occur in brain associated with Alzheimer's disease. This paper proposes an automated image processing based approach for the identification of AD from MRI of the brain. The proposed approach is novel in a sense that it has higher specificity/accuracy values despite the use of smaller feature set as compared to existing approaches. Moreover, the proposed approach is capable of identifying AD patients in early stages. The dataset selected consists of 85 age and gender matched individuals from OASIS database. The features selected are volume of GM, WM, and CSF and size of hippocampus. Three different classification models (SVM, MLP, and J48) are used for identification of patients and controls. In addition, an ensemble of classifiers, based on majority voting, is adopted to overcome the error caused by an independent base classifier. Ten-fold cross validation strategy is applied for the evaluation of our scheme. Moreover, to evaluate the performance of proposed approach, individual features and combination of features are fed to individual classifiers and ensemble based classifier. Using size of left hippocampus as feature, the accuracy achieved with ensemble of classifiers is 93.75%, with 100% specificity and 87.5% sensitivity.

## 1. Introduction

Alzheimer's disease (AD) is a progressive degeneration of the brain characterized by the accumulation of amyloid plaques and neurofibrillary tangles in brain tissues [1]. It is the major form of dementia with more than 35 million people all over the world. The probability of the disease doubles every five years after the age of 60 and it is estimated that by 2050 the figure will shoot up to 135 million. Death rate due to Alzheimer's has increased up to 68% since year 2000 [2]. It is a major public health issue of increasing importance as life expectancy of the population increases. Initially the disease affects an individual's capability to develop new memories but gradually other complications, like memory loss, orientation problems, poor judgment, inability to carry out routine tasks, and withdrawal from social activities, also arise. Currently the diagnostic criteria for AD is based on clinical and psychometric assessment tests like clinical dementia rate (CDR) and minimental state examination (MMSE) but the only definitive diagnosis of the disease can be done by autopsy of the brain. There is an immense need for the development of new methods for early identification of AD, and in this regard a number of brain imaging techniques facilitate providing noninvasive ways for visualization of brain atrophy [3–10]. Its earlier diagnosis in not only challenging but also crucial for further treatment.

Among brain imaging techniques, magnetic resonance imaging (MRI) is considered to be a surrogate to AD as it can measure structural changes in the brain [6]. MRI produces high resolution spatial images with minute abnormality detection property. It provides better visualization of internal structures of the brain as compared to CT scans. Moreover MRI involves no radiations, which could have possible side effects. Extensive studies have been performed for identification of AD using MRI. Comparative studies of various methods are presented in [11, 12] used for Alzheimer's disease identification and classification. Various classification techniques have been used for identification of structural changes in brain that can be possible indicators of the disease with the help of neuroimaging data [13–15]. The studies mentioned in literature are either based on region of interest (ROI) [16] or voxel-based morphometry (VBM) [17]. Most of the earlier ROI based methods are based on manual segmentation of the region of interest. The features extracted in these techniques are usually tissue densities [18], cortical thickness [19, 20], and volume and shape of hippocampus [21, 22]. The limitation of such technique is that they do not show high sensitivity and specificity in diagnosis of individuals because of the complex pathology of AD. In order to overcome the limitation of ROI based methods, VBM approach has been proposed, which uses entire pattern of brain atrophy instead of relying on ROI [23]. VBM can be successfully used for finding group differences but are of limited use when it comes to classifying individuals.

The recent trend in literature is towards the use of machine learning based high dimensional pattern classification methods [5, 24]. These techniques like support vector machines (SVM) help in the automated classification of MRI scans as either Alzheimer's disease or healthy controls [18, 25]. Such techniques do not rely on single region of interest, which may result in low specificity and sensitivity due to higher intersubject variability. The relationship among different brain regions is considered in these techniques resulting in higher discriminative power. The limitation of these techniques is the high dimensionality of the medical images as compared to little sample size.

Our proposed approach is based on image processing, machine learning, and pattern recognition techniques. Using image processing techniques, we preprocessed MRI images for meaningful analysis and feature extraction. The selected features are then forwarded to machine learning algorithms, which based on pattern recognition techniques classify MRI images into one of the possible categories. The strength of our approach lies in the fact that only few regions that are at the closest risk of atrophy are selected in the classification process and hence are reducing the computation time.

Rest of the paper is organized as follows: Section 2 presents the details of the dataset used and comprehensive working of our proposed approach, whereas Section 3 demonstrates the experiments done and Section 4 discusses the results achieved. The paper ends with the conclusion of our research work and possible future directions in Section 5.

## 2. Material and Methods

### 2.1. Data

In this research work we have chosen T1 weighted MRI scans of the brain because it provides good contrast of grey and white matter as compared to T2 weighted MRI. Our study is based on MRI scans from publicly available database of Open Access Series of Imaging Studies (OASIS) [26] which provides brain imaging data for analysis. The study has been performed for AD patients in mild stage. 416 brain scans are downloaded from OASIS including scans of healthy older controls, Alzheimer's disease patients, and healthy younger controls. Among these, 85 scans of older subjects are selected for this study based on the values of CDR, MMSE, and age of the subjects. 37 of 85 subjects are categorized as Alzheimer's disease patients based on CDR scores of 0.5 or 1 and MMSE score less than 25. The scores of CDR and MMSE indicate that patients are in mild stage of AD. A control sample of 48 individuals who remained unimpaired with CDR score equal to 0 and MMSE score higher than 28 matched for age and sex are identified and included in the study. Subject characteristics are shown in Table 1.

### 2.2. Proposed Approach

**Figure 1:**
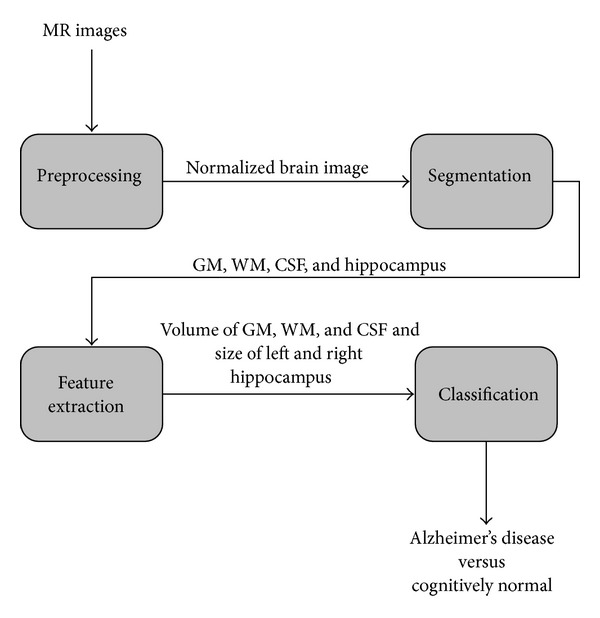
Block diagram of proposed approach.

The proposed approach consists of four phases, that is,* preprocessing*,* segmentation*,* feature extraction*, and* classification* of the MR images. The basic flow of work is shown in Figure 1.

**Figure 2:**
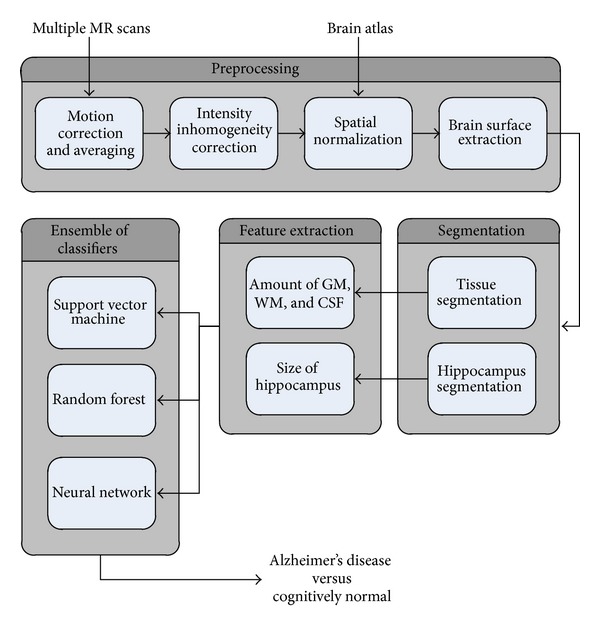
Detailed working of the proposed approach.

The first phase of our proposed approach prepares the input image (MRI) for further analysis. Second phase segments the image into different regions. Features are extracted in the third phase. The fourth and final phase classifies the image as belonging to a normal subject or Alzheimer's disease patient. The detailed working of the proposed approach is presented in Figure 2.

#### 2.2.1. Phase 1: Preprocessing

Any image processing algorithm like segmentation and feature extraction relies significantly on the quality of the images. The quality of MR images deteriorates either during its acquisition process or afterwards. The process of MRI acquisition may incorporate certain artifacts. For example noise problem and intensity inhomogeneity. To remove these and to optimize the image for further analysis and evaluation, a number of preprocessing steps are required. Image preprocessing can significantly increase the visual reliability of the image. It involves a set of techniques which enhances or eradicates certain details of the image in order to efficiently process it for further analysis. For our proposed approach the set of preprocessing includes motion correction and averaging, intensity inhomogeneity correction, spatial normalization, and brain surface extraction. The details of these steps are given below.

*(1) Phase 1.1: Motion Correction and Averaging.* MRI acquisition is a time demanding process with patients in completely still state. Unfortunately the voluntary or involuntary motion caused by patients may incorporate as a limiting factor in MRI exams. In order to remove this artifact we need motion correction algorithm. The simple method for motion correction is to take a number of repetitive scans in a single session and then calculate the average of all scans. In this way signal-to-noise ratio is increased.

(*2) Phase 1.2: Intensity Inhomogeneity Correction.* During the acquisition of MRI, due to the possible inhomogeneity of magnetic field or magnetic susceptibility variation in the subject, intensity labels that are assigned to tissue classes are not uniform. This intensity inhomogeneity can be seen as a smooth shading effect in the image which may result in poor segmentation and feature extraction. Any image processing algorithm that works with image intensity as a feature can greatly reduce performance due to intensity inhomogeneity. Fortunately this artifact known as gain field or bias field can be corrected using a number of algorithms. For our work, we have used the technique developed by [36] and available as BrainSuit tool. For bias field correction, the default values of BrainSuit typically provide improved results. The bias estimate range is set from 0.5 to 1.5 and spline stiffness is set to 0.0001. Other parameters are initialized as histogram radius to 12, sample spacing to 16, and control point spacing to 64.

(*3) Phase 1.3: Spatial Normalization.* In brain MRI analysis it is quite useful to coregister the brain image to a standard template brain. Because brain scans may differ in size and shape for individual subjects, wrapping these to same template will help in identification of the anatomical structures. In our approach we used most widely used brain template, that is, Talairach and Tournoux coordinate system [37]. Spatial normalization enables us to compare results across subjects and across different studies. It involves estimating a set of parameters that describe the transformation so that the input image is best fitted to the standard template brain image. In our work, default parameters of Talairach and Tournoux are used.

*(4) Phase 1.4: Brain Surface Extraction.* Our work is mainly concerned with WM, GM, and CSF. Unfortunately the intensities of these tissues may overlap with other regions of the head like bone and skin, and so forth. Therefore, there is a strong chance that the presence of these nonbrain voxels in MR image may reduce the reliability of identifying interested brain regions. For this purpose we require that nonbrain voxels be trimmed off the MR image. Brain surface extraction is a preprocessing step in which nonbrain tissue is removed from the MRI [38]. The parameters used for brain surface extraction are diffusion iterations, diffusion constant, and edge constant. These parameters are initialized to 3, 25, and 0.64. The erosion size is set to 1.

#### 2.2.2. Phases 2 and 3: Segmentation and Feature Extraction

Neuroimaging serves best in early detection of Alzheimer's disease as they hold necessary information to distinguish between healthy controls and Alzheimer's disease patients. But the major concern here is the huge data size of neuroimages. In order to classify these images by the classifier, the computational time is enormous. Moreover not all information in the images is required for the classification purposes as most of the information is irrelevant. For this purpose feature extraction is performed to find more relevant and discriminative features [39], in order to classify images more efficiently. In recent literature a vast variety of features has been extracted from MR images for the identification of AD. These features are voxel based [14, 18, 40], vertex based [20, 41], or ROI based features [21, 29]. Features that we have used in proposed approach are volume of GM, WM, and CSF and size of hippocampus. These selected features are affected in the earliest stages of AD. Hence the contribution of presented research is towards identification of AD patients in early stages, using a smaller feature set which results in lower computational expense.

Details of the features that have been extracted from MR scans are given below.

**Figure 3:**
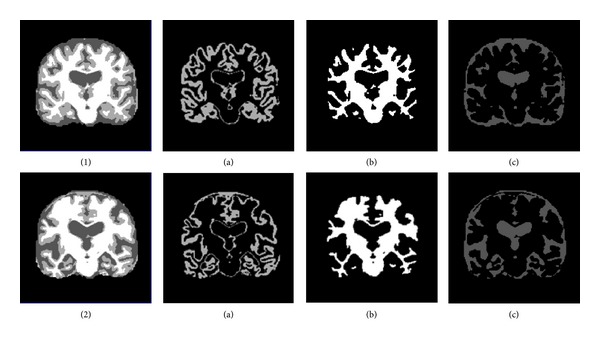
Shows CN in (1) and AD in (2). Segmented tissues are shown as (a) grey Matter (b) white matter, and (c) CSF.

**Figure 4:**
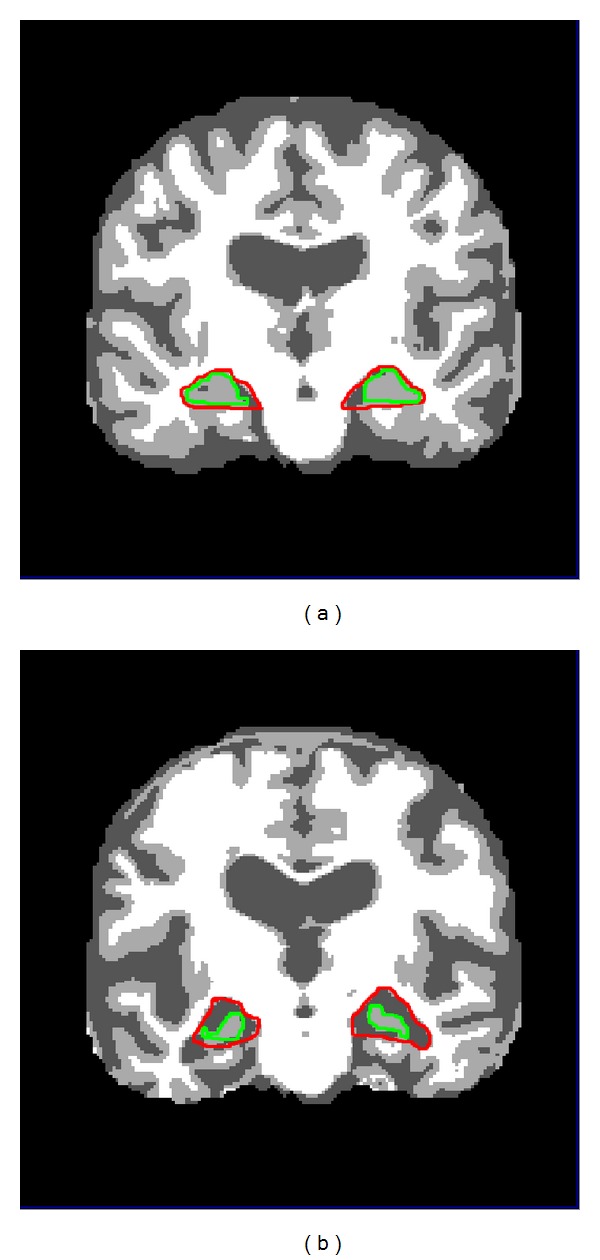
Brain of (a) CN and (b) AD. The estimated location of left and right hippocampus regions are shown in red, whereas actual hippocampus is shown in green.

*(1) Volume of GM, WM, and CSF.* Changes in GM, WM, and CSF volumes in whole brain or in specific regions may represent presence or severity of the disease. A number of studies have shown reduction in GM due to brain atrophy in Alzheimer's disease. We have investigated the volumes of different tissue classes, that is, GM, WM, and CSF in each slice of MRI. In order to obtain the tissue volumes we segmented preprocessed MR images into GM, WM, and CSF using FSL software package (freely available at http://www.fmrib.ox.ac.uk/fsl). Intensity threshold range “thresh” is used to describe GM. Regions below this threshold are CSF and above this threshold are WM. The volumes of these tissues have been calculated using the following equations:

(1) $$\begin{array}{c} \text{Volum}{\text{e}}_{\text{WM}}=\overset{n}{\underset{\text{slice}=1}{\sum }}\overset{x}{\underset{i=1}{\sum }}\overset{y}{\underset{j=1}{\sum }}f\left(i,j\right)>\text{thresh},\\ \text{Volum}{\text{e}}_{\text{GM}}=\overset{n}{\underset{\text{slice}=1}{\sum }}\overset{x}{\underset{i=1}{\sum }}\overset{y}{\underset{j=1}{\sum }}f\left(i,j\right)==\text{thresh},\\ \text{Volum}{\text{e}}_{\text{CSF}}=\overset{n}{\underset{\text{slice}=1}{\sum }}\overset{x}{\underset{i=1}{\sum }}\overset{y}{\underset{j=1}{\sum }}f\left(i,j\right)<\text{thresh}, \end{array}$$

where *f*(*i*, *j*) represents intensity of a pixel in a single slice of MRI. In Figure 3, reduction of grey matter in case of AD can be seen clearly.

(*2) Hippocampal Size.* Hippocampus is a structure in medial temporal lobe of the brain that is responsible for memory formation. It is affected in the earliest stages of Alzheimer's disease [42]. With the disease progression the size of hippocampus shrinks, which could be used as a potential biomarker for the detection of Alzheimer's disease. Atrophy measurement of hippocampus is commonly used for identification, progression, or even prediction of the disease. In literature much work has been done to measure the atrophy of hippocampus for probable identification of Alzheimer's disease. But still due to the complex structure of hippocampus, its segmentation is not an easy task and requires more attention to be paid. The process of hippocampus atrophy measurement from MRI imaging involves a number of steps including its segmentation. But the process faces a number of difficulties due to the presence of complex surrounding structures, low intensity differences between different structures, and absence of well-defined contours. For our proposed approach we chose coronal reformats of MRI because they are perpendicular to hippocampus and help us in hippocampus area calculation. It can be seen in Figure 4 that in case of Alzheimer's disease the hippocampal region contains relatively lower amount of grey matter as compared to normal subjects.

**Figure 5:**
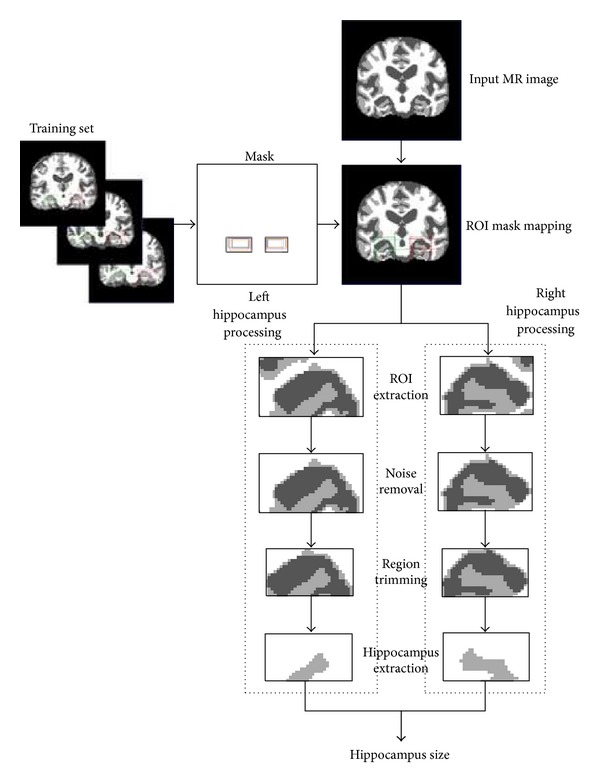
Proposed approach flow for hippocampus size calculation.

Our proposed approach, as shown in Figure 5, consists of ROI mask mapping, ROI extraction, noise removal, region trimming, hippocampus extraction, and hippocampi size calculation. As it was discussed earlier that hippocampus is surrounded by complex structures and has low intensity differences and no well-defined contours, so for its segmentation we have developed an ROI mask for estimation of the region including hippocampus. The rectangular ROI mask has been calculated by performing manual segmentation of hippocampus region on half of the images from available dataset which serves as a training set. From this training set it was found that in a spatially normalized 498 × 498 coronal MRI slice, the left and right hippocampi are bounded in rectangular regions formed by coordinates (130, 300) and (225, 360) for left hippocampus and (280, 300) and (375, 360) for right hippocampus. The estimated mask was mapped on the remaining half of images of dataset to test its correctness and it was discovered that it works well with 97% accuracy in segmenting the ROI including hippocampi.

The estimated ROI mask is mapped on new MRI slice and accordingly ROI is extracted from the MRI slice. The extracted ROI is one that includes hippocampal regions but it may also include some unnecessary surrounded structures which we call “noise.” The next step is to remove those unwanted structures or noise from the image. Our noise removal algorithm is based on the size of objects in the image. Smaller objects are removed by morphologically opening the binary image, while keeping the largest object which includes the hippocampus.

After noise removal region trimming is performed on the image, which cut down the border regions that are not part of the hippocampus. After region trimming, the image includes hippocampus in high intensity values and CSF in low intensity values on a white background. High intensity values representing hippocampal region can now be easily separated from the image in order to calculate its area. The calculated areas of left and right hippocampi are now used as features to be fed to the classifiers. In normal controls the values for area of left and right hippocampi are higher as compared to the same values for Alzheimer's disease patients.

#### 2.2.3. Phase 4: Classification

Three different types of classifiers, that is, SVM, MLP, and J48 are used to evaluate the accuracy of classification using different type of features. In addition to these classifiers, we also used ensemble of these classifiers to verify the enhanced accuracy rate.

*(1) Ensemble of Classifiers.* Ensemble of classifiers is a set of base classifiers that classify a new instance based on the voting of base classifiers [43]. The idea behind combining classifiers is to enhance the accuracy rate and it has been proved that accuracy rate of ensemble of classifiers is higher than the accuracy rate of individual base classifiers. The base classifiers must be diverse in nature for the ensemble classifier to outperform than base classifiers [44].

The ensemble classification technique that we have adopted is based on majority voting. Each base classifier's result is used as a vote, and ensemble decision class is one that has majority votes from base classifiers. The base classifiers that we have used in ensemble classifier are SVM, MLP, and J48. Consider

(2) $$\begin{array}{c} \text{Ensemble}\_\text{Class}=\{\begin{array}{cc} 1, & \text{if}\;\overset{n}{\underset{i=1}{\sum }}\text{Base}\_\text{Classifier}\_\text{Class}>\frac{n}{2},\\ -1, & \text{otherwise}, \end{array} \end{array}$$

where “1” represents Alzheimer's disease class, “−1” represents normal subject's class, “Base Classifier” is one of SVM, MLP, and J48, and “*n*” is the total number of base classifiers.

(*2) Support Vector Machines (SVM).* Support vector machines are one of the supervised multivariate classifiers [45]. SVM work by finding a hyperplane that best separates the two data groups. SVM are trained with training data in *n*-dimensional training space after which test subjects are classified according to their position in *n*-dimensional feature space. SVM have already been used for neuroimaging data [18, 46]. For our work, we have used SVM equipped with an RBF kernel. As the number of selected features is small, so RBF kernel performs better than linear kernel. The hyperparameters of SVM, that is, a regularization constant *C* and a kernel hyperparameter *γ*, are optimized using cross validation.

(*3) Multilayer Perceptron (MLP).* Multilayer perceptron is one of the supervised classifier. They are feed forward neural networks that work on back propagation algorithm for training. It usually consists of three or more layers, that is, an input layer, an output layer, and one or more hidden layers. MLP is used in situations where no algorithmic solution exists or algorithmic solution is too complex to be defined. MLP learns through training how input data is transformed into desired output. MLP is very popular for pattern recognition and interpolation. MLP is used for Alzheimer's disease detection from MRI and other types of imaging modalities [47, 48]. Initially the network is constructed with varying number of hidden layers and neurons/layer and it is learnt that 2 hidden layers and 3 neurons per layer perform better than others. The learning rate of network is set to 0.3.

*(4) Decision Tree (J48).* Decision tree is a machine learning based classification model that classifies an instance based on the attributes of the available data. C4.5 is a decision tree algorithm to create univariate trees [49]. Its working is based on the fact that while creating the tree recursively, it places that attribute at the root which has the highest information gain. J48 is WEKA's implementation of C4.5 algorithm that we used for classification [50]. C4.5 algorithm has already been used for Alzheimer's disease identification with different modalities [51]. For our proposed work, we have used the default value, used by most of the standard work, for the confidence factor parameter of J48 which is 0.25.

## 3. Results

To measure the performance of our proposed approach we have used three different types of classifiers (SVM, MLP, and J48) as well as an ensemble of these classifiers that works on majority voting. Ten-fold cross validation strategy is applied for the evaluation of our scheme. The purpose of choosing cross validation is to optimize the hyperparameters of the classifiers. Moreover, cross validation is used in problems where we want generalization to independent datasets, so a predictive model can be constructed.

First individual features, that is, volume of GM, volume of WM, volume of CSF, area of left hippocampus, and area of right hippocampus are fed to SVM, MLP, and J48. Then combinations of these features are applied to individual classifiers. First combination of features used is volume of GM, WM, and CSF. Second combination of features comprises areas of left and right hippocampi. Finally all five features are combined for the classification purpose. The results of the classification are provided in Table 2.

## 4. Discussion

**Figure 6:**
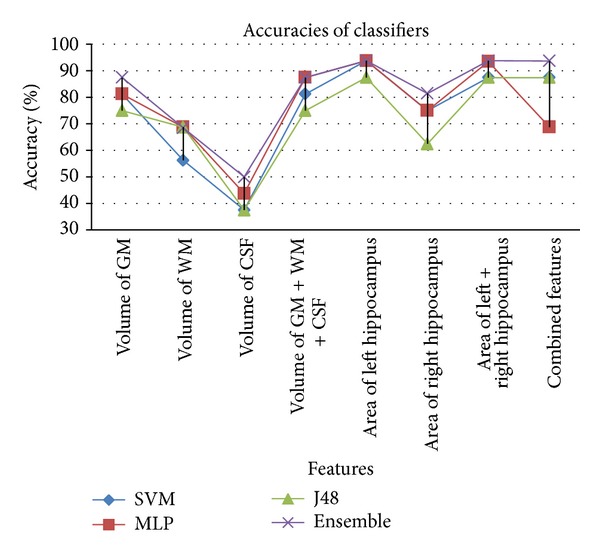
Comparison of % accuracies of classifiers based on different feature.

**Figure 7:**
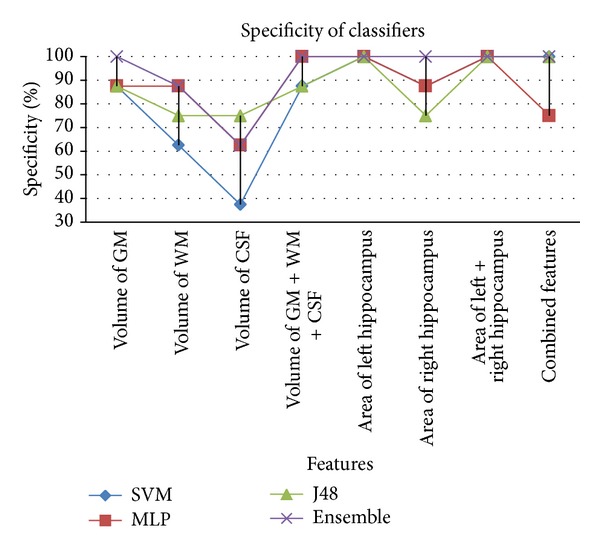
Comparison of % specificity achieved from classifiers based on different feature.

**Figure 8:**
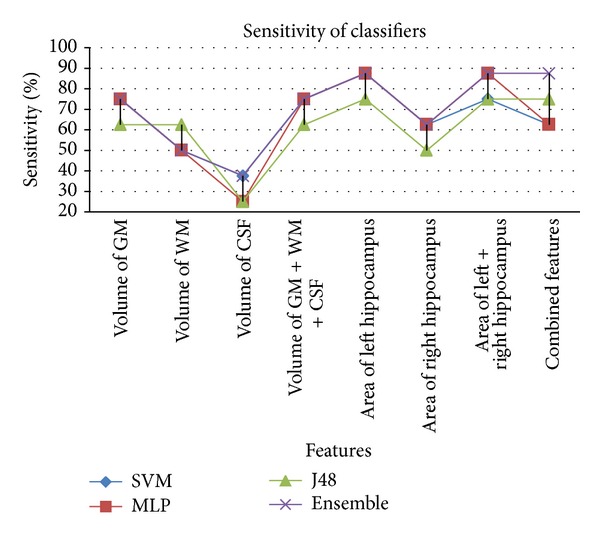
Comparison of % sensitivity achieved from classifiers based on different feature.

The ensemble based classifier performs better than individual classifiers in most of the cases (see Table 2). Features that provided better results are volume of GM and area of left hippocampus. It can also be seen that volume of WM and volume of CSF as features do not contribute towards satisfactory results. The results show that when using individual features SVM and multilayer perceptron perform better as compared to J48. But when combination of all features is taken into account then SVM and J48 achieve better accuracy rate as compared to multilayer perceptron. Among features used, left hippocampus area provides better classification rate on all classifiers. Comparing GM, WM, and CSF, it is evident that GM outperforms WM and CSF. Similarly, combination of GM, WM, and CSF gives better accuracy than individual features when using multilayer perceptron. From these results, it can be concluded that the ensemble of classifiers is a good option for the overall classification between Alzheimer's disease subjects and healthy controls due to its superior accuracy rate compared to its base classifiers. We have been able to achieve higher specificity/accuracy rates as compared to existing approaches (see Table 3) for identification of Alzheimer's patient in mild stage. Comparable results have been achieved with a smaller feature set, which contributed highly in reducing the computational time. Comparison of accuracy, specificity, and sensitivity of all classifiers with individual features as well as combined features is presented in Figures 6, 7, and 8, respectively.

In Figure 6, it can be seen that ensemble classifier's accuracy rate is higher than the individual classifier's accuracy rate. J48 performs worst for almost all types of features. Features that provide highest accuracy are “area of left hippocampus,” “area of left + right hippocampus,” and when all features are combined.

A comparison of specificity rates is shown in Figure 7. Again ensemble classifier provides higher rates of specificity as compared to SVM, MLP, and J48. Features that provide higher specificity rates are “volume of GM + WM + CSF”, “area of left hippocampus”, “area of right hippocampus”, “area of left + right hippocampus” and when all features are combined with ensemble classifier.

When comparing sensitivities of classifiers using different features, it is observed that ensemble classifier provides the highest sensitivities, whereas J48 provides the lowest sensitivities rates. This is depicted in Figure 8.

## 5. Conclusion 

In this research work we have investigated a new approach for the automated classification of Alzheimer's disease from MRI scans. We have presented a computer based automated methodology that will assist and help practitioners in diagnosis of AD. The proposed approach is based on the extraction of two types of features after preprocessing and segmentation of the images. We have used four classification models to evaluate our proposed approach based on individual features as well as a combination of all features. Using machine learning methods, the images are classified as Alzheimer's disease or normal subject. Classifiers that have been used are SVM, multilayer perceptron, J48, and ensemble of classifiers based on majority voting. Efficient and reliable results are achieved; that shows the effectiveness of our proposed approach. Comparison of our results shows that while considering individual features, left hippocampus achieves the highest accuracy with all three classifiers as well as ensemble of classifiers. When a combination of all features is used, SVM and J48 perform better than MLP.

The comparison of proposed approach with existing approaches (see Table 3) shows that the proposed approach has best specificity values as compared to that of existing approaches. The proposed approach has an overall accuracy value comparable to existing approaches despite the fact that we have used smaller feature set. Existing approaches have used the whole GM/WM volumes or larger ROIs while we have used only one ROI (left hippocampus) and GM volume (numeric value only).

Future perspectives of this research can take into account multiple modalities as well as genetic data to further improve the accuracy rate of classification.

## Figures and Tables

**Table 1:** Demographics of the participants included in the research.

Group	AD	NC
Number of subjects (*n*)	37	48
Male	12	10
Female	25	38
Years of education, mean ± S.D.	15.60 ± 3.54	16.82 ± 3.13
Age, mean ± S.D.	79.38 ± 5.95	79.44 ± 6.99
Cognitive scores		
CDR, mean ± S.D.	0.73 ± 0.25	0 ± 0
MMSE, mean ± S.D.	20.79 ± 2.58	29.52 ± 0.5

**Table 2:** Results of the classification.

Features	SVM			MLP			J48			Ensemble of classifiers		
	ACC%	SPE%	SEN%	ACC%	SPE%	SEN%	ACC%	SPE%	SEN%	ACC%	SPE%	SEN%
Volume of GM	81.25	87.5	75	81.25	87.5	75	75	87.5	62.5	87.5	100	75
Volume of WM	56.25	62.5	50	68.75	87.5	50	68.75	75	62.5	68.5	87.5	50
Volume of CSF	37.5	37.5	37.5	43.75	62.5	25	37.5	75	25	50	62.5	37.5
Volume of GM + WM + CSF	81.25	87.5	75	87.5	100	75	75	87.5	62.5	87.5	100	75
Area of left hippocampus	93.75	100	87.5	93.75	100	87.5	87.5	100	75	93.75	100	87.5
Area of right hippocampus	75	87.5	62.5	75	87.5	62.5	62.5	75	50	81.5	100	62.5
Area of left + right hippocampus	87.5	100	75	93.5	100	87.5	87.5	100	75	93.75	100	87.5
Combined features	87.5	100	62.5	68.75	75	62.5	87.5	100	75	93.75	100	87.5

ACC: accuracy, SPE: specificity, SEN: sensitivity.

**Table 3:** Comparison of proposed approach with existing approaches.

Approach	Features	Classifier	Accuracy		Specificity	Sensitivity
Ye et al., 2008 [27]	ROI and voxel based tensor	RKDA	—		89.50	95.00
		SVM	—		85.00	94.50

Long and Wyatt, 2010 [28]	WM	Quick shift clustering	94.67	96		—
	GM		97.33		—	

Kloppel et al., 2008 [6]	GM	SVM	95.6		94.1	97.1

Zhang et al., 2011 [29]	GM volume (93 ROIs)	SVM	86.2		86.3	86

Casanova et al., 2013 [30]	GM-voxel	RLR	87.1		88.9	84.3

Chu et al., 2012 [31]	GM-voxel	SVM	84.3		—	—

Cuingnet et al., 2011 [12]	GM-voxel	SVM	88.6		95	81

Vemuri et al., 2008 [32]	GM + WM + CSF voxels	SVM	—		86.0	86.0

Wee et al., 2013 [19]	Correlation and ROI based morphological features	SVM	92.35		94.31	90.35

Teipel et al., 2007 [33]	GM + WM	Logistic regression	83		78	88

Westman et al., 2013 [34]	Regional MRI measures (259 features)	OPLS	91.5		92.9	89.8

Hinrichs et al., 2009 [14]	GM-voxels	LP boosting	82		80	85

Wolz et al., 2011 [13]	Hippocampus volume, tensor-based morphometry, cortical thickness, manifold learning based features	LDA	89		93	85

Liu et al., 2014 [35]	GM-voxel	Hierarchical fusion	92		93	90.9

Proposed approach	ROI (left hippocampus)	Ensemble of classifiers	93.75	90.5	100	87.5
	Volume of GM		87.5		100	75
